# Draft Genome Sequence of *Photorhabdus temperata* Strain Meg1, an Entomopathogenic Bacterium Isolated from *Heterorhabditis megidis* Nematodes

**DOI:** 10.1128/genomeA.01273-14

**Published:** 2014-12-11

**Authors:** Sheldon G. Hurst, Shimaa Ghazal, Krystalynne Morris, Feseha Abebe-Akele, W. Kelley Thomas, Usama M. Badr, Mona A. Hussein, Mohamed A. AbouZaied, Kamal M. Khalil, Louis S. Tisa

**Affiliations:** a University of New Hampshire, Durham, New Hampshire, USA; b Applied Microbial Genetics Laboratory, Genetics and Cytology Department, Genetic Engineering & Biotechnology Division, National Research Centre, Dokki, Cairo, Egypt; c Department of Pests and Plant Protection, Agricultural Division, National Research Centre, Dokki, Cairo, Egypt; d Department of Microbiology, Faculty of Science, University of Ain Shams, Abbassia, Cairo, Egypt

## Abstract

*Photorhabdus temperata* strain Meg1 is an entomopathogenic bacterium that forms a symbiotic association with *Heterorhabditis* nematodes. We report here a 4.9-Mbp draft genome sequence for *P. temperata* strain Meg1, with a G+C content of 43.18% and containing 4,340 candidate protein-coding genes.

## GENOME ANNOUNCEMENT

Members of the genus *Photorhabdus* maintain two distinct life styles as insect pathogens and in symbiotic relationship with the entomopathogenic *Heterorhabditis* nematodes (for reviews, see references 1–7). The life cycle of *Photorhabdus* and its nematode host *Heterorhabditis* is best described as a cyclic association that begins and ends with infective juvenile nematodes (IJs). The IJs, which carry a monoculture of *Photorhabdus* within the anterior region of the intestine (8, 9), actively seek and infect insect hosts. Once inside the insect, the nematodes regurgitate the bacteria into the hemolymph (8). The bacteria multiply inside the host and release highly virulent toxins (10, 11), resulting the death of the insect death in <48 h. To provide nutrients for both the bacteria and nematodes, the bacteria secrete extracellular enzymes that aid in breaking down insect tissue and also generate essential growth factors for the growth and development of the nematode. The growth and development of *Heterorhabditis* nematodes have an obligate requirement for their specific bacterial symbiont (12). To prevent secondary invaders and putrefaction of the insect carcass, the bacteria release antibiotics (13, 14). After several days of feeding, the nematodes and bacteria reassociate and leave in search of a new insect host.

Based on molecular analysis, the genus *Photorhabdus* is divided into three bacterial species: *Photorhabdus luminescens*, *Photorhabdus temperata*, and *Photorhabdus asymbiotica* (15, 16). Here, we present a draft genome sequence for *P. temperata* strain Meg1, which was isolated from *Heterorhabditis megidis* nematodes (17). This bacterial strain is used to propagate axenic nematodes. The *Heterorhabditis bacteriophora* nematodes grow and reproduce normally on Meg1 bacteria, but the resulting surface-sterilized IJ nematodes do not retain Meg1 bacteria and are therefore axenic (18). Because of this property, we chose *P. temperata* strain Meg1 for sequencing to provide an understanding of its symbiotic association.

The draft genome of *P. temperata* subsp. *temperata* strain Meg1 was generated at the Hubbard Genome Center (University of New Hampshire, Durham, NH) using Illumina technology (19) techniques. A standard Illumina shotgun library was constructed and sequenced using the Illumina HiSeq 2000 platform, which generated 44,504,248 reads (260-bp insert size) totaling 4,450.4 Mbp. The Illumina sequence data were assembled using CLC Genomics Workbench (version 6.5.1) and AllPaths-LG (version r41043) (20). The final draft assembly contains 127 contigs, with an *N*_50_ of 86 kb. The total size of the genome is 4.9 Mbp, and the final assembly is based on 3,841 Mb of Illumina draft data, providing an average 778.9× coverage of the genome.

The high-quality draft genome of *P. temperata* strain Meg1 was resolved to 127 contigs consisting of 4,931,142 bp, with a G+C content of 43.18%. The assembled *P. temperata* strain Meg1 genome was annotated via the Integrated Microbial Genomes (IMG) platform developed by the Joint Genome Institute, Walnut Creek, CA, USA (21), and resulted in 4,340 candidate protein-coding genes.

### Nucleotide sequence accession numbers.

This whole-genome shotgun project has been deposited at DDBJ/EMBL/GenBank under the accession no. JGVH00000000. The version described in this paper is version JGVH01000000.
